# Identification of High-Risk Atypical Meningiomas According to Semantic and Radiomic Features

**DOI:** 10.3390/cancers12102942

**Published:** 2020-10-12

**Authors:** Darius Kalasauskas, Andrea Kronfeld, Mirjam Renovanz, Elena Kurz, Petra Leukel, Harald Krenzlin, Marc A. Brockmann, Clemens J. Sommer, Florian Ringel, Naureen Keric

**Affiliations:** 1 Department of Neurosurgery, University Medical Centre, Johannes Gutenberg University Mainz, 55131 Mainz, Germany; mirjam.renovanz@med.uni-tuebingen.de (M.R.); Elena.Kurz@unimedizin-mainz.de (E.K.); Harald.Krenzlin@unimedizin-mainz.de (H.K.); Florian.Ringel@unimedizin-mainz.de (F.R.); Naureen.keric@unimedizin-mainz.de (N.K.); 2 Department of Neuroradiology, University Medical Centre, Johannes Gutenberg University Mainz, 55131 Mainz, Germany; Andrea.Kronfeld@unimedizin-mainz.de (A.K.); Marc.Brockmann@unimedizin-mainz.de (M.A.B.); 3 Division of Neuro-Oncology, University Medical Centre Tubingen, 72016 Tübingen, Germany; 4 Institute of Neuropathology, University Medical Centre, Johannes Gutenberg University Mainz, 55131 Mainz, Germany; Petra.Leukel@unimedizin-mainz.de (P.L.); Clemens.Sommer@unimedizin-mainz.de (C.J.S.)

**Keywords:** atypical meningioma, survival, radiomics, prediction

## Abstract

**Simple Summary:**

More than 50% of atypical meningiomas recur within 5 years. Identification of high-risk tumors might be helpful for pre-operative planning. The aim of our retrospective study was to assess the value of radiomic and semantic magnetic resonance imaging (MRI) characteristics for the prediction of tumor relapse. Our findings suggest that the semantic characteristic of cystic component and the radiomic feature of cluster prominence are associated with tumor recurrence. A combination of semantic and radiomic characteristics is a promising tool for identifying patients with high-risk atypical meningiomas.

**Abstract:**

Up to 60% of atypical meningiomas (World Health Organization (WHO) grade II) reoccur within 5 years after resection. However, no clear radiological criteria exist to identify tumors with higher risk of relapse. In this study, we aimed to assess the association of certain radiomic and semantic features of atypical meningiomas in MRI with tumor recurrence. We identified patients operated on primary atypical meningiomas in our department from 2007 to 2017. An analysis of 13 quantitatively defined radiomic and 11 qualitatively defined semantic criteria was performed based on preoperative MRI scans. Imaging characteristics were assessed along with clinical and survival data. The analysis included 76 patients (59% women, mean age 59 years). Complete tumor resection was achieved in 65 (86%) cases, and tumor relapse occurred in 17 (22%) cases. Mean follow-up time was 41.6 (range 3–168) months. Cystic component was significantly associated with tumor recurrence (odds ratio (OR) 21.7, 95% confidence interval (CI) 3.8–124.5) and shorter progression-free survival (33.2 vs. 80.7 months, *p* < 0.001), whereas radiomic characteristics had no predictive value in univariate analysis. However, multivariate analysis demonstrated significant predictive value of high cluster prominence (hazard ratio (HR) 5.89 (1.03–33.73) and cystic component (HR 20.21 (2.46–166.02)) for tumor recurrence. The combination of radiomic and semantic features might be an effective tool for identifying patients with high-risk atypical meningiomas. The presence of a cystic component in these tumors is associated with a high risk of tumor recurrence.

## 1. Introduction

Atypical meningiomas (World Health Organization (WHO) grade II) comprise an intermediate group of tumors between benign (WHO grade I) and rare malignant (WHO grade III) meningiomas [1]. Following the latest WHO revision of histological criteria, the proportion of atypical meningiomas increased from about 5% to more than 20% [2]. Tumor recurrence is observed in 30–60% of patients within 5 years after surgical resection, and the extent of resection emerges as the most consistent risk factor in retrospective studies [3,4]. Additional risk factors include age, the use of postoperative radiation and tumor location [4,5,6]. Some histological and molecular markers have been described and correlated with a poorer outcome and a shorter progression-free survival (PFS) [7,8]. These advances are leading to a better understanding of the biological behavior of meningiomas. However, there is no ubiquitous availability of molecular and genetic diagnostic procedures. Therefore, there is a strong need for defined risk stratification factors. Unambiguous radiological criteria that could fill that void have not been established yet.

Radiomics, a method that processes imaging data according to various mathematical algorithms, allows for extraction of data that cannot be detected by the human observer. These algorithms are promising for the characterization of tumor type and behavior of some cancers [9,10]. Neuro-oncologists have successfully applied radiomics in the survival assessment of glioblastoma [11]. Recent studies on the use of radiomic analysis for meningiomas suggest that they can be used to estimate WHO grades [12,13,14], with close to 90% accuracy [15]. Radiomics could also be applied for the preoperative differentiation of meningioma subtypes [16]. Furthermore, the combination of both radiomic and visual identification of macroscopic (or semantic) characteristics has allowed for meningioma classifications based on WHO grading [12]. Therefore, we sought to evaluate the usefulness of a combined approach to identify the atypical meningiomas at high risk of recurrence, which could help clinicians optimize pre-operative planning of the tumor removal and further treatment.

## 2. Results

### 2.1. Patients

A total of 76 patients (59% women) with atypical meningioma were identified in our database. Table 1 shows the major demographic and tumor characteristics of the patients. The mean age was 58.7 years, range 12–81 years. The majority of tumors were localized on the convexity and in the falx. In 65 (86%) cases, the tumors could be resected completely (Simpson grade 1 or 2). Postoperative radiation was used to treat 22 (29%) patients, and tumor recurrence was observed in 17 (22%) cases. Mean follow-up time was 41.6 months (range 3–168 months). In 43 (57%) cases, each brain invasion, as well as 4–19 mitotic figures, were histologically identified, while in 26 (34%) cases, both criteria overlapped. Immunohistochemical analysis revealed no loss of the trimethylation of histone H3K27 (H3K27me3). We found a mutation in the telomerase reverse transcriptase (TERT) promotor in 3 (4%) cases, and one of these patients presented with a tumor relapse after 31 months.

Preoperative MRI data used for extraction of radiomic features were available from 51 (67%) patients, and semantic features could be described for 55 (72%) patients. No preoperative imaging could be found in the database for 21 (28%) patients. The major characteristics of the patients with radiomic and semantic data were similar to those of the entire patient population (Table 1).

### 2.2. Semantic and Radiomic Features

**Figure 1:**
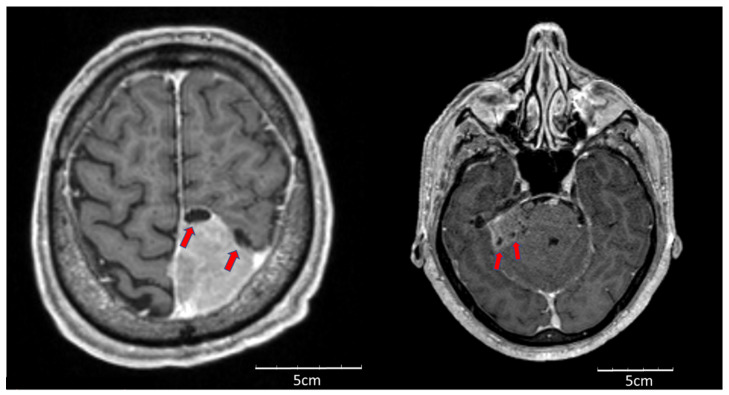
Cystic component within intracranial meningioma.

Using univariate logistic regression analysis of semantic features, we found that the cystic component (Figure 1) was significantly associated with tumor relapse (odds ratio (OR) 21.7, 95% confidence interval (CI) 3.8–124.5). Univariate analysis of radiomic features showed a predictive trend for cluster prominence (area under the curve (AUC) 0.663, *p* = 0.10). Therefore, an arbitrary threshold of 91,000 was calculated for cluster prominence based on the Youden’s index; however, it did not reach statistical significance (OR 3.8 (95% CI 0.93–15.5). These two features were included in a multivariate analysis, which found a significant association with tumor recurrence (Table 2). We did not observe a correlation between cystic component and cluster prominence.

**Figure 2:**
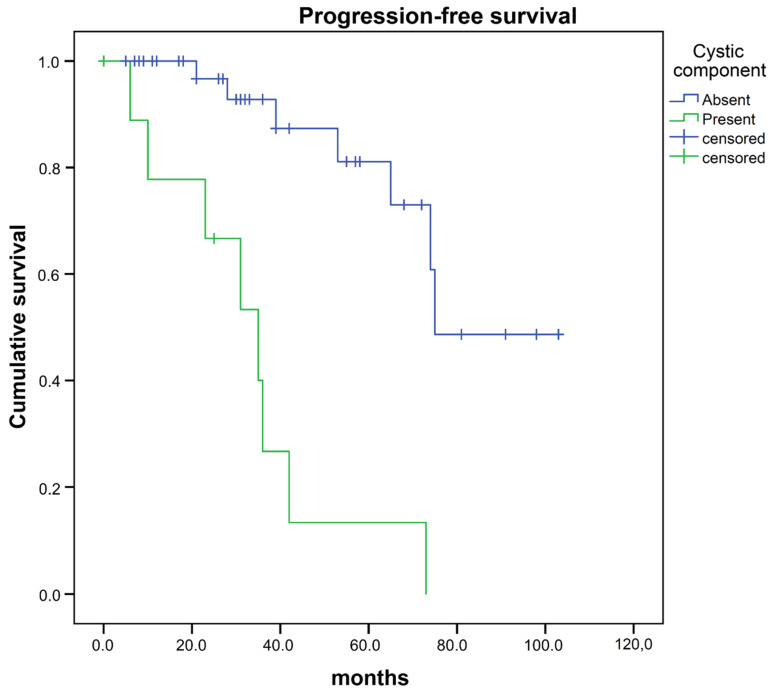
Progression-free survival after surgery of atypical meningiomas with vs. without “cystic component”.

We then analyzed the association of progression-free survival with radiomic and semantic features and clinical characteristics. For this purpose, resected tumors were classified as complete (Simpson grade 1 + 2) or incomplete (Simpson grade 3 + 4). Univariate analysis found a significant association between progression-free survival and completeness of resection OR 0.35 (95% CI 0.12–0.99), as well as with the feature cystic component OR 9.77 (95%CI 3.14–30.41) (Table 3). Cystic component was identified in 9 meningiomas and was associated with significantly shorter PFS (mean PFS 33.2 vs. 80.7 months, *p* < 0.001 (log-rank test), Figure 2). Postoperative radiation was not a statistically significant factor for PFS OR 2.12 (95%CI 0.81–5.53). PFS association with telomerase reverse transcriptase (TERT) promoter mutation and the loss of trimethylation of histone H3K27 (H3K27me3) could not be evaluated due to the low rate of identified TERT promotor mutations and no case of loss H3K27me3. In a multivariate analysis, only cystic component remained significantly associated with PFS (hazard ratio (HR) 22.74 (95%CI 4.29–120.66)) (Table 4).

## 3. Discussion

In this study, we evaluated the radiomic and semantic criteria of atypical meningiomas with regard to the prediction of tumor recurrence. We found that tumors presenting with cystic component or high cluster prominence were associated with tumor recurrence. Cystic component was strongly associated with a shortened PFS. Univariate analysis found a significant association of the extent of tumor resection with PFS. Other clinical parameters, including postoperative radiation, did not improve the PFS. Even though the completeness of resection was not consistently significant in multivariate analysis, residual tumor is a well-established risk factor for meningioma recurrence [4,17,18]. Because of conflicting results from retrospective studies, the effect of postoperative radiation remains controversial [4,17,19,20,21,22], while no prospective data is available yet.

Some meningioma characteristics such as the presence of tumor fringes (suggesting invasion of the brain tissue) and intra-tumoral hypodense areas were recognized as signs of atypia or malignancy early on [23]. Coroller et al. [12] found that 8 radiomic and 4 semantic characteristics could be used for prediction of WHO grade, and showed the potential for identifying more aggressive tumors. However, WHO grading does not completely reflect tumor behavior. For example, stratification according to apparent diffusion coefficient (ADC) and Simpson grading outperformed WHO grading for the prediction of tumor recurrence [24], meaning that the integration of available data is needed for patients at high-risk within WHO grades.

Among radiomic characteristics analyzed, high cluster prominence demonstrated a predictive value for tumor recurrence in a multivariate analysis. It is a feature which is sensible to the areas of similar intensity within the tumor. The association of cluster prominence with more aggressive tumors is in accordance with the finding of Coroller et al. [12], as it has shown a classifying value (AUC 0.65) for atypical meningiomas, together with 3 other radiomic features (mean intensity, low-intensity small area emphasis and difference entropy). Even though cluster prominence showed a prognostic value for tumor recurrence in a multivariate analysis, there was no significant association with PFS. The only significant feature associated with PFS in univariate and multivariate analyses was cystic component.

Several studies suggested that cystic component is a predictor for a more aggressive tumor behavior [17,25,26]. A study of 75 patients with 59 benign and 16 atypical or malignant meningiomas, which evaluated 6 imaging features potentially associated with aggressive tumor behavior, found that only intra-tumoral cystic change and extracranial tumor extension were significantly associated with high tumor grade [25]. Another study of patients with atypical meningiomas found that heterogeneous enhancement, cyst formation and peritumoral edema were associated with tumor recurrence [17]. A systematic review of 35 studies demonstrated that cyst formation and tumor heterogeneity were associated with higher WHO grade; however, it was mostly not related to progression [26]. Epigenetic modification on the level of histones, namely the loss of H3K27me3 [27], as well as TERT promoter mutation [8] were associated with increased risk of meningioma recurrence. In our study cohort, all cases showed a retained trimethylation of histone H3K27. Furthermore, only in 4% of cases was a TERT promotor mutation \identified. Consequently, a clinical impact of these molecular markers could not be evaluated.

Other radiological features also appear to show an association with higher tumor grade. Apparent diffusion coefficient (ADC) has been shown to be correlated with WHO grade in a multicenter study [28]. Another group was able to classify patients according to WHO grade using the combination of Fluid-attenuated inversion recovery (FLAIR), T1 contrast-enhanced and ADC sequences [14]. Altogether, these results indicate that newly developed analysis algorithms are promising tools for a more nuanced tumor stratification. Machine learning algorithms that were implemented to predict meningioma grade on preoperative MRIs have been shown to be highly accurate [29].

The results of our study must be interpreted with caution because of its retrospective nature, small patient sample and limited number of radiomic criteria used. However, we think that the combination of radiological data together with known clinical data and established risk factors should be effective for the identification of high-risk patients. If verified, this approach might lead to a machine learning-based software solution for MRI interpretation and be helpful for the selection of the optimal treatment strategy for patients for whom observation and surgery can be considered.

## 4. Materials and Methods

### 4.1. Patient Sample and Study Design

We identified patients with meningiomas who underwent surgery in our department from 2007 to 2017 in our database. The inclusion criteria were: histological confirmation of primary (i.e., not manifesting the progression of WHO grade I meningiomas) atypical intracranial meningiomas and at least one postoperative follow-up ≥3 months after surgery. Patients with history of neurofibromatosis and previous brain radiation were excluded from the study. In addition to standard histological work-up for meningioma (Hematoxylin and eosin (HE), Reticulin stain, Ki67-immunohistochemistry), the tumor samples were screened for Telomerase reverse transcriptase (TERT) promoter mutations as well as loss of trimethylation of histone H3K27 (H3K27me3). Radiomic analysis was performed on preoperative MRI scan with contrast-enhanced (CE) T1 sequences. In case of insufficient quality (movement artefacts, distortions, inability to import into the software, etc.), the imaging was not used for extraction of radiomic characteristics. However, it could be used for analyzing semantic characteristics in some cases. Among patients with multiple MRI scans, MR images acquired nearest the date of surgery were selected.

### 4.2. Radiomic and Semantic Analysis

Tumor volume was extracted from contrast-enhanced T1 sequences by three-dimensional (3D) Slicer software Version 4.8.1 (www.slicer.org) using a manual segmentation [30,31]. Radiomic features were extracted by an in-house Matlab-script (Matlab R2017b, The MathWorks, Inc. (Natick, MA, USA)) based on the tools and methods described by Vallières et al. [32] and Coroller et al. [12]. Because radiomics entails a large number of quantitative imaging features, we reduced redundancy for the comparison of prognostic features. We chose our characteristics according to the study by Coroller et al. [12], to classify meningiomas into WHO grades based on their semantic and radiomic features, as these characteristics have shown the most potential to define the tumor aggressiveness. A total of 13 quantitatively defined radiomic characteristics were used along with wavelet and Laplacian of Gaussian filters (Table 4 and Table S1). Semantic analysis was done by two experienced neurosurgeons independently. In case of disagreement, the certain feature was reviewed and agreed upon. Eleven semantic features in total were defined qualitatively (as a presence or absence of certain characteristics, Table 4 and Table S1).

### 4.3. H3K27me3 Immunohistochemistry

Immunohistochemistry was performed on 4 µm thick routinely processed formalin-fixed and paraffin-embedded tissue sections. After dewaxing, antigen retrieval using EnVision FLEX Target Retrieval Solution, high pH (Dako #S2368 Glostrup, Denmark), was performed. Afterwards, endogenous peroxidase was blocked by peroxidase blocking solution (DAKO, Glostrup, Denmark) and sections were stained with anti-Tri-Methyl-Histone H3 (Lys27) primary antibody 1:200 (Cell Signaling Technology, Leiden, The Netherlends) using an immunostainer (Dako Autostainer Plus, DAKO, Glostrup, Denmark). Immunoreactivity was visualized by the universal immuno-enzyme polymer method (Nichirei Biosciences, Tokyo, Japan). Finally, sections were developed in diaminobenzidine (Lab Vision Cooperation, Fermont, CA, USA). Omission of the primary antisera in a subset of control slides resulted in no immunostaining at all. Analysis of nuclear H3K27me3 immunostaining was performed by an experienced neuropathologist (CJS).

### 4.4. Sequencing of Genomic DNA from Formalin-Fixed, Paraffin-Embedded Meningioma Samples

Tumor areas of 76 meningioma paraffin sections were scratched off according to corresponding HE-staining and DNA preparation was performed using a QIAamp DNA FFPE Tissue Kit (Qiagen, Hilden, Germany). Polymerase chain reaction (PCR) reactions were performed in 25 µL reaction mixture using FastStart™ Taq DNA Polymerase-Kit (Merck KGaA, Darmstadt, Germany), with forward (5′-CCG TCC TGC CCC TTC ACC-3′) and reverse (5′-GGG CCG CGG AAA GGA AG-3′) TERT-specific primers. PCR products were directly sequenced using the CEQ Dye terminator cycle sequencing reagents (Beckman Coulter, Brea, CA, USA) with the Beckman CEQ 8800 capillary gel electrophoresis system. Sequence results were analyzed by Chromas, DNA sequencing software (Technelysium Pty Ltd., South Brisbane, Australia).

### 4.5. Statistics

**Figure 3:**
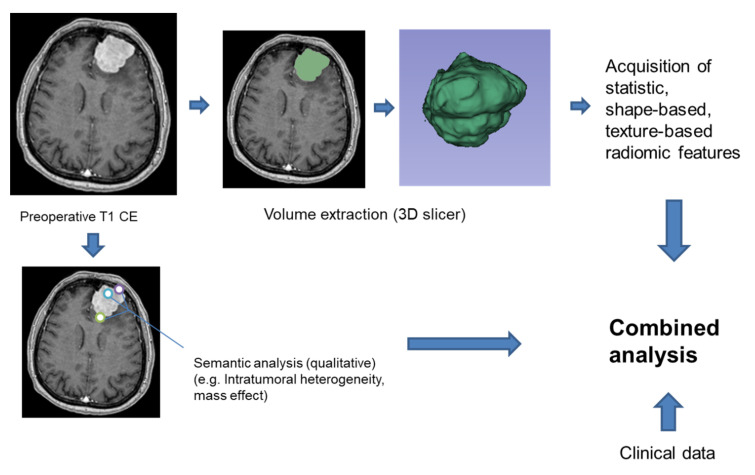
Workflow of the study.

The data were assessed along with clinical information that included the following characteristics: patient age, gender, completeness of tumor removal and PFS (Figure 3). The effect of postoperative radiation was evaluated. Univariate and multivariate regression analysis as well as receiver operator characteristics (ROC) curve analysis were performed using SPSS software, Version 26.0 (IBM Corporation, Armonk, NY, USA), and used to evaluate the tumor features and clinical data in association with patient outcome.

### 4.6. Ethical Approval

The ethics committee of Rhineland-Palatinate, Germany, reviewed and approved this study (837.476.17 (11310)). All procedures performed were in accordance with the ethical standards of the institutional and national research committee and with the 1964 Helsinki declaration and its later amendments or comparable ethical standards.

## 5. Conclusions

The combination of radiomic with semantic features might be a helpful tool for identifying patients with high-risk atypical meningiomas. The presence of a cystic component in these tumors is associated with a high risk of tumor recurrence. A prospective study including a higher number of patients can validate these initial results and respectively lead to risk stratification in the clinical setting.

## Figures and Tables

**Table 1:** Patient characteristics.

Characteristic	All Patients	Radiomic Data Available	Semantic Data Available
N (%)	76 (100)	51 (67.1)	55 (72.4)
Age (SD)	58.7 (13.8)	59.1 (14.1)	58.8 (13.9)
Women, %	59.2	64.7	65.5
Tumor localization, %			
Convexity/falx	59.2	58.8	61.8
Scull base	39.5	39.2	36.4
Ventricular	1.3	2	
Simpson Grade, %			
1°	71.1	66.7	68.5
2°	14.5	15.7	14.5
3°	7.9	7.8	9.1
4°	5.3	7.8	7.3
Postoperative radiation, %	28.9	31.4	30.9
Tumor relapse, N (%)	17 (22.4)	11 (21.6)	15 (27.3)

SD—standard deviation.

**Table 2:** Univariate and multivariate analysis of semantic characteristics and selected radiomic feature (cluster prominence) for tumor recurrence.

Univariate Analysis	Odds Ratio	95%CI
Intra-tumoral heterogeneity	0.19	0.02–1.61
Multifocality	0	
Midline shift	2.75	0.75–10.11
Sinus invasion	0.62	0.12–3.30
Necrosis Hemorrhage	2.71	0.78–9.38
Mass effect	4.67	0.54–40.11
Cystic component†	21.74	3.79–124.54
Bone invasion	2.12	0.64–7.08
Hyperostosis	2.04	0.60–6.91
Spiculation	1.18	0.31–4.45
Edema	1.18	0.31–4.45
High cluster prominence *	3.79	0.93–15.47
**Multivariate Analysis**	**Hazard Ratio**	**95%CI**
Cystic component ^†^	20.21	2.46–166.02
High cluster prominence ^†,^*	5.89	1.03–33.73

* cluster prominence more than 91,000, ^†^ *p* < 0.05, CI—confidence interval.

**Table 3:** Association of progression-free survival (PFS) with clinical, semantic and radiomic characteristics.

Univariate Analysis	Odds Ratio	95%CI
Completeness of resection ^†^	0.35	0.12–0.99
Tumor localization ^‡^	1.62	0.59–4.47
Gender	0.44	0.17–1.17
Age older than 65 years	1.36	0.50–3.68
Postoperative radiation	2.12	0.81–5.53
Cystic component ^†^	9.77	3.14–30.41
High cluster prominence *	1.15	0.32–4.09
**Multivariate Analysis**	**Hazard Ratio**	**95%CI**
Completeness of resection	1.27	0.31–5.24
Cystic component ^†^	22.74	4.29–120.66
High cluster prominence *	1.35	0.34–5.47

* cluster prominence more than 91,000, ^‡^ Falx and convexity vs. Scull base, ^†^ *p* < 0.05.

**Table 4:** Radiomic, semantic and clinical data used in the analysis. More detailed explanation of radiomic and semantic features is provided in Supplementary Materials Table S1.

Radiomic Characteristics	Semantic Characteristics	Clinical Characteristics
Mean	Intra-tumoral heterogeneity	Age
Median	Multifocality	Gender
Minimum	Midline shift	Tumor localization
Skewness	Sinus invasion	Completeness of resection
Spherical Disproportion	Necrosis/Hemorrhage	Postoperative radiation
Cluster Prominence	Mass effect	Progression-free survival
Difference Entropy	Cystic component	
Inverse Difference Normalized	Bone invasion	
Run Length Non-uniformity	Hyperostosis	
Short-Run Low Gray-Level Emphasis	Spiculation	
High-Intensity Large Area Emphasis	Edema	
Low-Intensity Large Area Emphasis		
Low-Intensity Small Area Emphasis		
**Filters**		
Wavelet		
Laplacian of Gaussian		

## Supplementary Materials

The following are available online at https://www.mdpi.com/2072-6694/12/10/2942/s1, Table S1: Description of radiomic and semantic features used in tumor analysis.
