# Supplementary material for: Identification of High-Risk Atypical Meningiomas According to Semantic and Radiomic Features
Source: Cancers (Basel). 2020 Oct 12;12(10):2942. doi: 10.3390/cancers12102942 (PMC7599676; doi:10.3390/cancers12102942)
Supplement: Supplementary file 1 [file cancers-12-02942-s001.pdf]

cancers

Supplementary material

Identification of High-Risk Atypical Meningiomas According to Semantic and Radiomic Features

Darius Kalasauskas, Andrea Kronfeld, Mirjam Renovanz, Elena Kurz, Petra Leukel, Harald Krenzlin, Marc A. Brockmann, Clemens J. Sommer, Florian Ringel and Naureen Keric

Table S1. Description of radiomic and semantic features used in tumor analysis.

**Radiomic characteristics**	
Mean	Mean voxel intensity value
Median	Median voxel intensity value
Minimum	Minimal voxel intensity value
Skewness	Measure of the assimetry of a probability distribution of the voxel intensity value
Spherical Disproportion	The difference of the tumor shape to the sphere with a similar volume
Cluster Prominence	Sensitive to the areas of similar intensity
Difference Entropy	Complexity of the pattern
Inverse Difference Normalized	Sensitive to the homogenity of the tumor
Run Length Non-uniformity	Sensitive to heterogeneity
Short Run Low Gray-Level Emphasis	Measures the heterogeneity sensitive to low intensity pattern
High Intensity Large Area Emphasis	Sensitive to flat zones with high intensity voxels (e.g. hemorrhage)
Low Intensity Large Area Emphasis	Sensitive to flat zones with low intensity voxels (e.g. necrosis)
Low Intensity Small Area Emphasis	Sensitive to small flat zones with low intensity voxels
**Semantic characteristics**	
Intratumoral heterogeneity	Heterogeneity throughout the tumor
Multifocality	Non-contiguous growth of tumor
Midline shift	Shift of brain past midline
Sinus invasion	Presence of venous sinus invasion
Necrosis / Hemorrhage	Presence of necrosis or hemorrhage
Mass effect	Presence of brain shift due to the tumor
Cystic component	Fluid filled cysts within the tumor
Bone invasion	Appearance of tumor invading the skull
Hyperostosis	Bony overgrowth adjacent to the tumor
Spiculation	Irregularities of tumor shape and border
Edema	Brain swelling due to the tumor

© 2020 by the authors. Licensee MDPI, Basel, Switzerland. This article is an open access article distributed under the terms and conditions of the Creative Commons Attribution (CC BY) license (http://creativecommons.org/licenses/by/4.0/).
